# Impact of dapagliflozin on bone mineral metabolism in non-diabetic patients with chronic kidney disease: a randomized, double-blind, placebo-controlled study

**DOI:** 10.1093/ckj/sfaf384

**Published:** 2025-12-09

**Authors:** Mohamed Elshayeb, Ahmed Abdulgalil, Amr El-Husseini, Muhammed Elhadedy, Samir Sally, Huda Refaie, Wael Mortada, Kareem Nabieh, Mohamed Sobh

**Affiliations:** Department of Nephrology, Dialysis and Transplantation Unit, Urology and Nephrology Center, Mansoura University, Mansoura City, Egypt; Mansoura Nephrology and Dialysis Unit (MNDU), Mansoura University, Mansoura City, Egypt; Division of Nephrology & Bone and Mineral Metabolism, University of Kentucky, Lexington, KY, USA; Department of Nephrology, Dialysis and Transplantation Unit, Urology and Nephrology Center, Mansoura University, Mansoura City, Egypt; Department of Nephrology, Dialysis and Transplantation Unit, Urology and Nephrology Center, Mansoura University, Mansoura City, Egypt; Department of Radiology, Urology and Nephrology Center, Mansoura University, Mansoura City, Egypt; Clinical Chemistry Lab, Urology and Nephrology Center, Mansoura University, Mansoura City, Egypt; Clinical Chemistry Lab, Urology and Nephrology Center, Mansoura University, Mansoura City, Egypt; Department of Nephrology, Dialysis and Transplantation Unit, Urology and Nephrology Center, Mansoura University, Mansoura City, Egypt

**Keywords:** bone mineral density, bone turnover markers, chronic kidney disease, dapagliflozin, SGLT2i

## Abstract

**Background:**

Sodium–glucose co-transporter 2 inhibitors have demonstrated renoprotective effects in patients with CKD. However, concerns remain regarding their potential effect on bone mineral metabolism. This study investigated the impact of dapagliflozin on bone health in non-diabetic patients with CKD.

**Methods:**

This randomized, double-blind, placebo-controlled trial enrolled 100 non-diabetic adults with CKD and an estimated glomerular filtration rate (eGFR) of 25–75 ml/min/1.73 m^2^. Participants were randomized 1:1 to receive either dapagliflozin 10 mg daily or placebo for 12 months and stratified by age and eGFR. Serum creatinine, eGFR, urinary protein:creatinine ratio, calcium:creatinine ratio and phosphorus:creatinine ratio were measured at baseline and after 12 months. Bone health was assessed at the same time points using bone formation markers [bone-specific alkaline phosphatase (BSAP), total procollagen type 1 N-terminal propeptide (P1NP)] and bone resorption markers [carboxy-terminal telopeptide of type I collagen (CTX-1), tartrate-resistant acid phosphatase 5b (TRAP-5b)]. Moreover, quantitative computed tomography (QCT) was used to assess volumetric bone mineral density (vBMD).

**Results:**

Participants had a mean age of 53.5 ± 11.1 years, with no significant baseline differences between groups. Dapagliflozin significantly reduced proteinuria compared with placebo (*P* = .012) without significantly affecting eGFR. Both groups experienced significant decreases in BSAP and TRAP-5b levels (*P* < .001), with no intergroup differences. P1NP remained stable in both groups. CTX-1 levels increased significantly in the placebo group (*P* = 0.032) but not in the dapagliflozin group without significant intergroup differences. No significant differences in vBMD or T-scores at any lumbar or total lumbar spine were observed between groups after 1 year.

**Conclusions:**

This exploratory randomized controlled trial did not demonstrate any meaningful impact of dapagliflozin on either bone turnover or QCT markers. While these findings provide reassuring preliminary evidence, larger studies are needed to definitively establish long-term bone safety of dapagliflozin in non-diabetic patients with CKD.

KEY LEARNING POINTS **What was known:**

- Sodium–glucose co-transporter 2 inhibitors (SGLT2i) have been proved to have a renoprotective effect in diabetic and non-diabetic CKD patients.
- Concerns have been raised regarding their effect on bone and mineral metabolism that may lead to an increase in the risk of fracture.
- The effect of SGLT2is is still debatable and underinvestigated, especially in non-diabetic patients with CKD.

**This study adds:**

- Compared with placebo, dapagliflozin significantly reduced proteinuria after 1 year, with preservation of the kidney function.
- One-year use of dapagliflozin did not negatively impact bone health, proved by stability of bone turnover markers and quantitative computed tomography results.
- Provides preliminary exploratory evidence showing no detectable bone harm signals of dapagliflozin use over 1 year, though larger studies are needed for definitive safety conclusions.

**Potential impact:**

- This study provides the first exploratory, prospective randomized controlled trial on bone effects of dapagliflozin in non-diabetic patients with CKD. These findings could contribute to future pooled and meta-analyses and call for longer-term trials on a greater number of patients.

## INTRODUCTION

Chronic kidney disease (CKD) is a major health problem, as ≈10% of the adult population have CKD [1]. CKD has high morbidity and mortality, particularly among individuals with diabetes and hypertension [2]. Several studies have demonstrated the effectiveness of sodium–glucose co-transporter 2 inhibitors (SGLT2is) in slowing CKD progression in both diabetic and non-diabetic patients [3, 4]. Their effect is not only dependent on glycaemic control but also on a variety of pleiotropic effects, including decreased intraglomerular pressure via an enhanced tubuloglomerular feedback mechanism, decreased transport of sodium and glucose over the proximal tubular cells, increased natriuresis and decreased systemic blood pressure [5].

CKD emerges as an independent risk factor for osteoporosis [6]. The impact of SGLT2is on the human skeleton is controversial. While some studies have shown that SGLT2is might have some detrimental impacts on the metabolism of minerals and bone health [7–9], others do not [10–12]. Furthermore, the potential negative impact of SGLT2is on bone is not clearly understood. They cause a mild increase in urinary calcium excretion secondary to osmotic diuresis, which increases tubular blood flow and reduces calcium reabsorption in the proximal tubules [13]. The increased urinary calcium excretion triggers a feedback increase in parathyroid hormone (PTH) secretion, which in turn acts to restore serum calcium balance by promoting bone resorption [14]. Moreover, the proximal renal tubule’s type II sodium–phosphate co-transporters increase phosphate reabsorption, causing the release of fibroblast growth factor 23 (FGF23) that suppresses 1,25-dihydroxyvitamin D synthesis, which reduces intestinal calcium absorption and subsequently increases the release of PTH [15, 16].

Notably, while some studies have shown that SGLT2is may affect bone turnover markers (BTMs) with increased carboxy-terminal telopeptide of type I collagen (CTX-1) and tartrate-resistant acid phosphatase 5b (TRAP-5b) levels, indicating increased bone resorption [11, 17, 18], others have revealed no impact on BTMs [19, 20]. Similarly, the impact of SGLT2is on bone mineral density (BMD) is also controversial. While canagliflozin use was associated with a decrease in BMD, particularly in hip and lumbar vertebrae [17], other studies did not demonstrate a deleterious effect of SGLT2is on BMD [10–12].

The debate extends to the risk of bone fractures with SGLT2i use. While some studies have suggested an increased fracture risk [8, 21], other studies have failed to confirm this finding [22].

We designed this prospective, randomized, double-blind, placebo-controlled trial to explore the potential effects of dapagliflozin on BTMs and volumetric bone mineral density (vBMD) in non-diabetic patients with CKD.

## MATERIALS AND METHODS

### Trial design and oversight

This is a prospective, single-centre, randomized, double-blind, placebo-controlled clinical trial conducted at Mansoura Urology and Nephrology Center, Mansoura City, Egypt from September 2022 to November 2023. The study aimed to evaluate the effect of dapagliflozin on bone mineral metabolism in non-diabetic patients with CKD. A total of 100 patients were enrolled and randomly assigned in a 1:1 ratio and stratified according to age and eGFR to receive either dapagliflozin (10 mg once daily) or a placebo. The trial was approved by the institutional review board (MD.22.02.600) and conducted in accordance with the principles of the Declaration of Helsinki. This study was also registered at ClinicalTrials.gov (NCT05735197) and all participants providing informed consent.

### Participants

**Figure 1:**
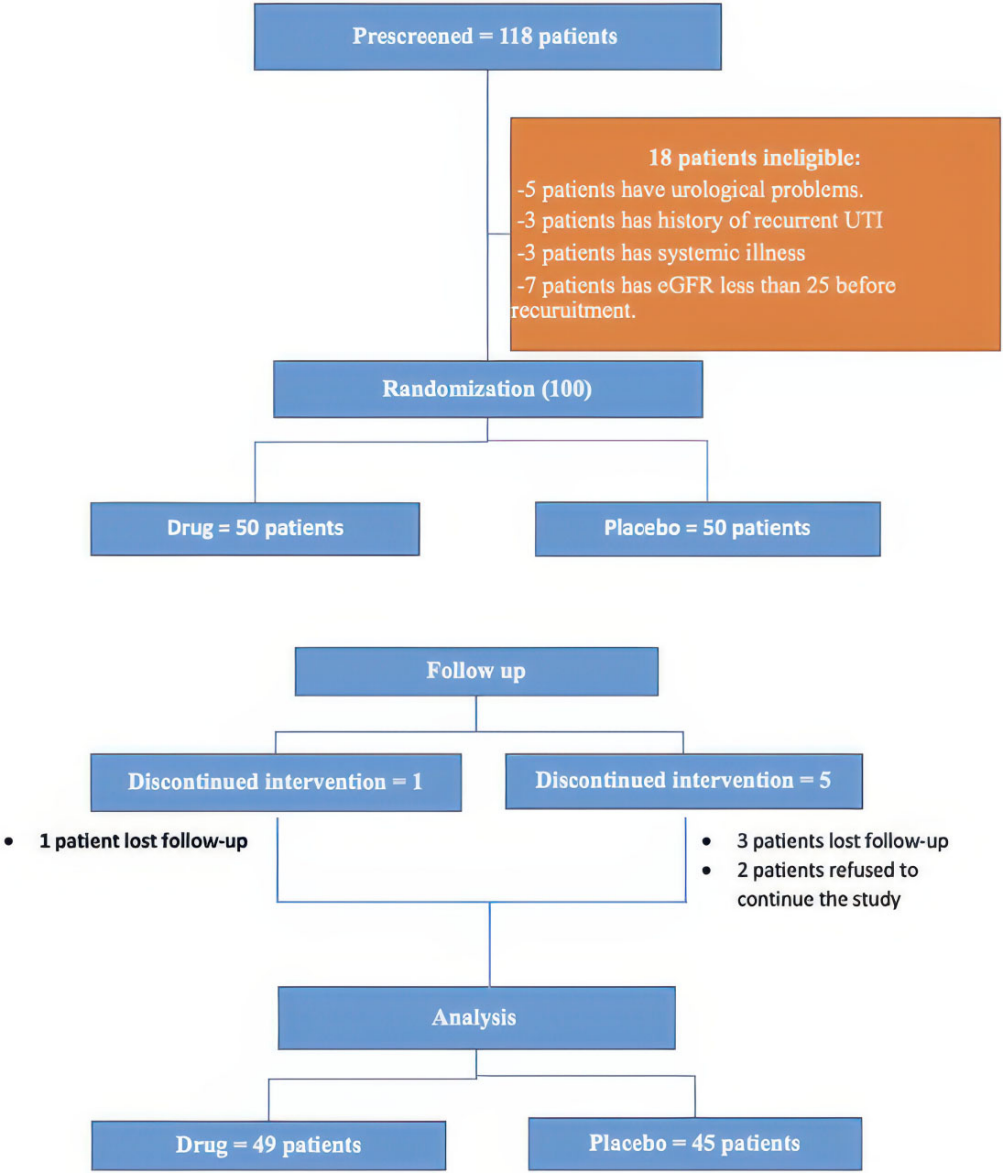
Participant flow diagram. This diagram describes the flow of participants throughout the study, including enrolment criteria, randomization and follow-up procedures.

Eligible participants were non-diabetic adults (≥18 years) with an eGFR of 25–75 ml/min/1.73 m^2^ with no evidence of acute kidney injury in the prior 3 months. Key exclusion criteria included patients with chronic active liver, respiratory diseases, infections (human immunodeficiency virus, hepatitis B virus, hepatitis C virus, tuberculosis), patients receiving SGLT2i or medications affecting bone metabolism (including immunosuppressive therapies) for >3 months prior to the study and pregnant or lactating women. Moreover, patients with a history of recurrent urinary tract infections, active malignancy or organ transplantation were also excluded (Fig. 1).

We selected an eGFR range of 25–75 ml/min/1.73 m^2^ to include patients with CKD stages G2–G4, the group most at risk for mineral bone disorder and disease progression. Only 10% of participants had an eGFR ≥60 ml/min/1.73 m^2^, limiting inclusion of near-normal kidney function. Randomization was stratified by baseline eGFR to ensure balanced kidney function severity across treatment arms, thereby strengthening internal validity.

#### Sample size calculation

The sample size was calculated to detect a clinically meaningful difference in bone turnover markers between the dapagliflozin and placebo groups. At the time of study design, no published data were available regarding the effect size of dapagliflozin on bone metabolism in patients with CKD. Consequently, the estimation was based on the best available evidence from canagliflozin trials, the only SGLT2i with reported changes in BTMs at that time. Among these markers, β-C-telopeptide (β-CTX) was the only parameter shown to respond measurably to SGLT2is. Therefore, data from the randomized study by Bilezikian *et al.* [17] were used to inform the anticipated effect size and variability for our calculation.

That trial reported ≈10–22% increases in CTX after 52 weeks of canagliflozin compared with placebo. Assuming a 20% relative between-group difference and a standard deviation (SD) of 30%, we calculated that 48 participants per arm would provide 90% power at a two-sided α of 0.05. Allowing for a 5–10% attrition rate, the target enrolment was set at 100 patients (50 per group). Calculations were performed using PASS version 15 (NCSS, Kaysville, UT, USA).

This approach was reasonable given the lack of dapagliflozin-specific data in CKD at the time. Dapagliflozin-related studies, such as Ljunggren *et al.* [19], have reported smaller and non-significant CTX changes with dapagliflozin, indicating that our original assumption may have been optimistic. Accordingly, our trial should be interpreted as exploratory and hypothesis-generating, providing preliminary evidence for future adequately powered clinical trials.

### Trial procedures

Patients were randomized using a computer-generated schedule stratified by age and eGFR. Both the dapagliflozin and placebo tablets were identical in appearance. Follow-up visits were conducted 1 month after randomization and every 3 months thereafter. At each visit, clinical examination was performed along with collecting blood and urine samples. Data on adverse events, adherence to medication and any additional treatments were also recorded. Serum and urinary biomarkers of metabolic bone disease were assessed [serum calcium, phosphorus, magnesium, intact parathyroid hormone (iPTH), urinary calcium:creatinine ratio, urinary phosphorus:creatinine ratio) and BTMs [procollagen type 1 N-terminal propeptide (P1NP), TRAP-5b, bone-specific alkaline phosphatase (BALP), CTX-1] and quantitative computed tomography (QCT) at baseline and after 1 year, with T-scores used to categorize bone health.

Serum creatinine, calcium, phosphorus, uric acid and magnesium were measured by automated technique using an ARCHITECT c4000 (Abbott, Abbott Park, IL, USA). eGFR was calculated using the Chronic Kidney Disease Epidemiology Collaboration 2021 equation. iPTH was measured by electrochemiluminescence immunoassay (ECLIA; Roche Elecsys PTH on a cobas e-601 analyzer; normal range: 16–65 pg/ml), with intra-assay coefficient of variability (CV) 0.7–2.4%, interassay CV 1.1–4.4% and estimated reference change value ≈5–12% depending on concentration. Urinary calcium:creatinine ratio and urinary phosphorus:creatinine ratio were calculated from spot urine measurements.

BTMs were determined using a commercial enzyme-linked immunosorbent assay (ELISA) kit (Chongqing Biases Co., Chongqing, China). Bone-specific alkaline phosphatase (BSAP) was quantified by sandwich ELISA (Biospes, Chongqing; catalogue BYEK3163; analytical range 50–600 pg/ml; sensitivity 5 pg/ml; intra-/interassay CV <8% and <10%, respectively). TRAP-5b was measured with an ELISA specific for the 5b isoform (IDS, Scottsdale, AZ, USA; analytical range 100–1000 pg/ml; sensitivity 10 pg/ml; intra-assay CV 4.7%, interassay CV 9.0%). TRAP-5b concentrations in this study are expressed as mass units (pg/ml) according to the Biospes ELISA kit. Other assays, such as the IDS kit, report TRAP-5b activity in enzymatic units (U/l), which are not directly interchangeable because conversion depends on the reference standard’s specific activity. To aid comparison, we provide the manufacturer’s reference range for healthy adults (2.5–10 ng/ml). Published enzymatic assays typically report 1.03–4.15 U/l in premenopausal women, which corresponds broadly to ≈2–8 ng/ml. Total P1NP and the resorption marker CTX-1 were determined on an automated electrochemiluminescence platform (Elecsys, Roche Diagnostics, Indianapolis, IN, USA). Total P1NP demonstrated a within-run CV of 2.1% and a between-run CV of 1.4–2.3% [2], while CTX-1 showed interlaboratory CVs consistently <7.6% across centres. All markers were expressed as mass concentrations (pg/ml or ng/ml) in accordance with manufacturer specifications.

BMD was assessed at baseline using QCT. Scans of the lumbar spine (L1–L5) were acquired using a CT scanner calibrated with solid calcium hydroxyapatite phantoms to ensure accurate BMD measurements. QCT analysis provided vBMD measurements (cm^3^) for trabecular bone at each vertebral level, which reflects metabolically active bone that is highly responsive to hormonal and metabolic disturbances. Given that trabecular bone is preferentially and earlier affected, lumbar QCT provides a sensitive means of detecting early skeletal changes in this population. Hip QCT was not performed due to equipment limitations. T-scores, representing the number of SDs from the mean BMD of young healthy adults, were also calculated.

### Statistical analysis

The statistical analysis was performed using R software (R Foundation for Statistical Computing, Vienna, Austria). Continuous variables were assessed for normality using the Shapiro–Wilk test. Normally distributed variables were expressed as mean ± SD and compared using Welch’s *t*-test, while non-normally distributed data were presented as median [interquartile range (IQR)] and analysed using the Mann–Whitney U test. Categorical variables were summarized as frequencies and percentages, with comparisons made using the chi-squared test or Fisher’s exact test.

Differences between the same group in pre- and post-intervention were analysed using the paired T-test and paired Wilcoxon signed-rank test for normally distributed and non-normally distributed data, respectively.

An analysis of covariance (ANCOVA) was conducted, adjusting for baseline values of key variables, such as eGFR and BTMs, to control for potential confounding factors. For non-normally distributed data or when ANCOVA assumptions were not met, quantile regression was used. *P*-values <.05 were considered statistically significant.

For multivariable analysis we employed the directed acyclic graph (DAG) methodology to identify minimal sufficient adjustment sets for confounding control (Supplementary Fig. 5). The DAG analysis indicated that adjusting for baseline eGFR was sufficient to eliminate confounding bias from backdoor paths. We performed two complementary multivariable analyses: DAG-based minimal models adjusting for baseline eGFR only and comprehensive clinical models adjusting for baseline eGFR, baseline values of each outcome variable, age, sex, weight change, baseline PTH, body mass index (BMI) and CKD aetiology to enhance precision and address clinical considerations.

## RESULTS

Baseline characteristics are presented in Table 1, which showed no statistically significant differences between the two groups regarding demographic data and biochemical parameters, apart from a higher urinary calcium:creatinine ratio in the dapagliflozin group (*P* = .039). The mean age of participants was 53.5 ± 11.1 years, with no significant difference between the dapagliflozin (53.1 ± 10.8 years) and placebo (54.1 ± 11.6 years) groups (*P* = .66). The distribution of sex was nearly identical, with 29% of female participants in both groups (*P* = .97). Similarly, no statistically significant differences were observed between the groups in terms of body weight (*P* = .28) or BMI (*P* = .46), indicating comparable anthropometric measurements. Key indicators of renal function, including serum creatinine and eGFR, were also well-matched between the groups at baseline. Importantly, no significant differences were found in markers of mineral metabolism, including serum calcium, phosphorus, magnesium and iPTH. Baseline urinary chemistry analysis revealed statistically similar protein:creatinine and phosphorus:creatinine ratios between the two groups (Table 1).

**Table 1:** Baseline characteristics of study participants.

Characteristics	Drug (*n* = 49)	Placebo (*n* = 45)	*P*-value^a^
Demographics			
Age (years), mean ± SD	53.08 ± 10.82	54.11 ± 11.62	.66
Female, *n* (%)	14 (29)	13 (29)	.97
Body weight (kg), mean ± SD	90.89 ± 15.10	87.72 ± 13.32	.28
BMI (kg/m^2^), mean ± SD	31.81 ± 4.63	31.00 ± 5.89	.46
Serum chemistry			
Creatinine (mg/dl), mean ± SD	1.77 ± 0.46	1.80 ± 0.44	.71
eGFR (ml/min), mean ± SD	44.75 ± 13.04	43.07 ± 10.46	.49
Intact PTH (ng/ml), median (IQR)	52.8 (39.6–89.9)	59.6 (46.5–76.1)	.86
Calcium (mg/dl), mean ± SD	9.32 ± 0.64	9.25 ± 0.64	.57
Phosphorus (mg/dl), mean ± SD	3.28 ± 0.76	3.14 ± 0.66	.37
Magnesium (mg/dl), mean ± SD	1.94 ± 0.22	1.93 ± 0.16	.73
Uric acid (mg/dl), mean ± SD	7.00 ± 1.68	6.57 ± 1.74	.23
Random blood sugar (mg/dl), mean ± SD	101.84 ± 16.24	104.87 ± 21.34	.44
Urinary chemistry, median (IQR)			
Protein:creatinine ratio (mg/mg)	0.83 (0.53–1.52)	0.95 (0.50–1.89)	.63
Calcium:creatinine ratio (mg/mg)	0.05 (0.03–0.08)	0.04 (0.02–0.06)	**.039**
Phosphorus:creatinine ratio (mg/mg)	0.42 (0.35–0.55)	0.46 (0.27–0.52)	.67
Medications, *n* (%)			
Oral calcium	29 (59.2)	26 (57.8)	˃.9
Active vitamin D	31 (63.6)	27 (60)	.833
Statin	22 (45)	24 (53.3)	.536
Uric acid–lowering agent	34 (69.4)	31 (68.9)	˃.9
Diuretic	9 (18)	9 (20)	.84

^a^Independent sample *t*-test, chi-squared test or Mann–Whitney test.

Bold values indicate statistically significant differences between groups (P < .05).

Table 2 presents the anthropometric, serum and urinary parameters after 12 months. A statistically significant reduction in both body weight and BMI was observed in the dapagliflozin group compared with the placebo group. Regarding kidney function, no significant differences were observed between the dapagliflozin and placebo groups in terms of serum creatinine or eGFR. However, a statistically significant reduction in the urinary protein:creatinine ratio was observed in the dapagliflozin group compared with the placebo group (*P* = .04). No statistically significant differences were detected between the dapagliflozin and placebo groups in serum levels of calcium, phosphorus, magnesium, iPTH or urinary calcium:creatinine and phosphorus:creatinine ratios after 12 months.

**Table 2:** Anthropometric, serum and urinary parameters after 12 months of treatment.

Characteristics	Drug	Placebo	*P*-value^a^
Body weight (kg), mean ± SE	87.06 ± 0.77	89.72 ± 0.81	**.04**
BMI (kg/m^2^), mean ± SE	31.19 ± 0.32	31.51 ± 0.34	**.005**
Serum chemistry			
Creatinine (mg/dl), mean ± SE	1.99 ± 0.06	1.89 ± 0.06	.71
eGFR (ml/min), mean ± SE	41.45 ± 1.50	44.08 ± 1.56	.23
Intact PTH (ng/ml), median (IQR)	70.2 (52.1–91.8)	74.4 (56.4–100.4)	.75
Calcium (mg/dl), mean ± SE	9.20 ± 0.06	9.09 ± 0.06	.24
Phosphorus (mg/dl), mean ± SE	3.35 ± 0.08	3.27 ± 0.08	.48
Magnesium (mg/dl), mean ± SE	2.04 ± 0.02	1.97 ± 0.02	.07
Uric acid (mg/dl), mean ± SE	6.37 ± 0.25	6.42 ± 0.26	.89
Random blood sugar (mg/dl), mean ± SE	102.26 ± 3.03	107.1 ± 3.16	.74
Urinary chemistry, median (IQR)			
Protein:creatinine ratio (mg/mg)	0.78 (0.45–1.32)	0.82 (0.46–1.87)	**.04**
Calcium:creatinine ratio (mg/mg)	0.039 (0.025- 0.057)	0.032 (0.021- 0.054)	.74
Phosphorus:creatinine ratio (mg/mg)	0.33 (0.27- 0.42)	0.32 (0.23- 0.40)	.57

SE: standard error.

^a^ANCOVA, quantile regression.

Bold values indicate statistically significant differences between groups (P < .05).

**Figure 2:**
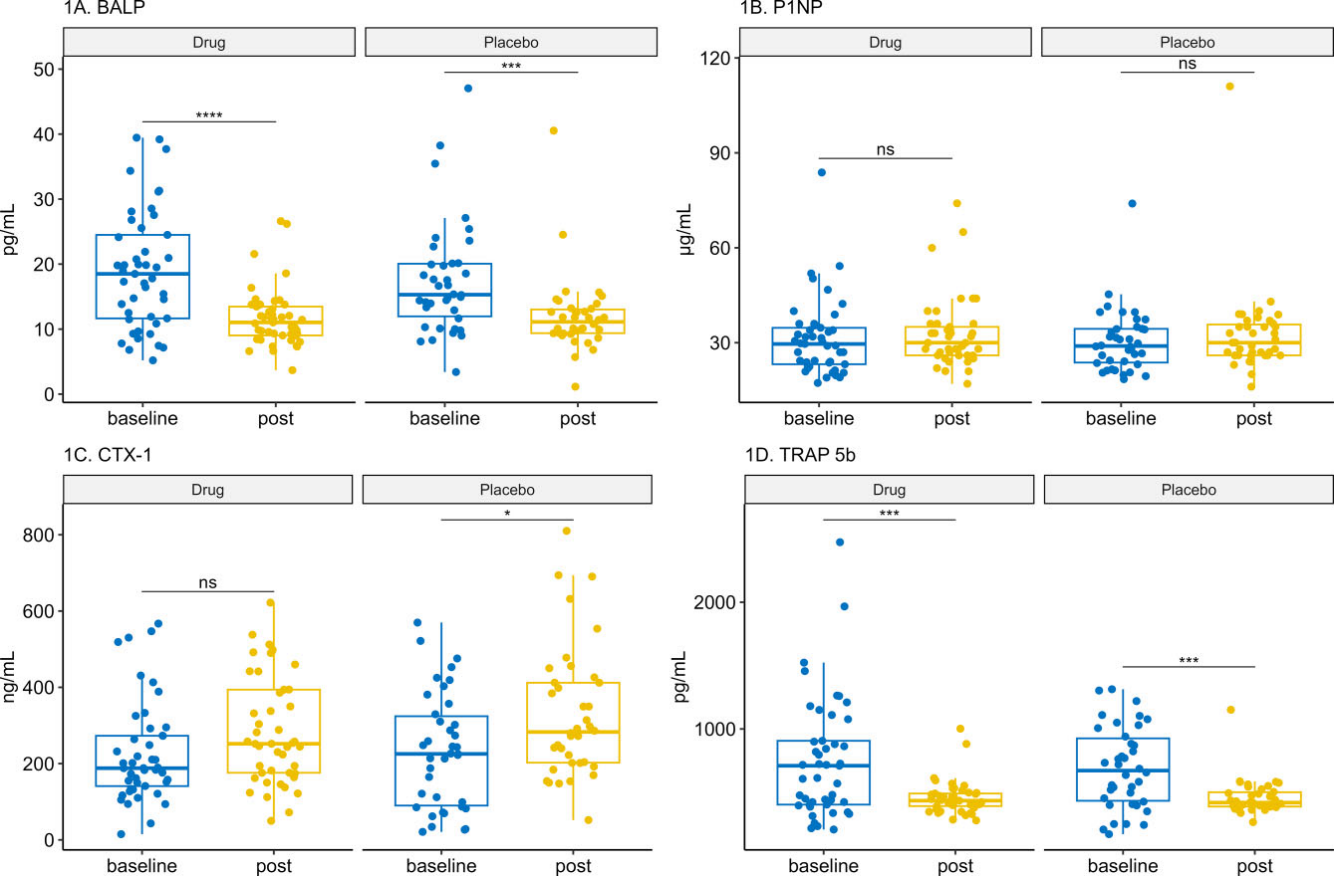
Changes in BTMs over 12 months. Box plots illustrate changes in BTMs from baseline to 12 months for both the dapagliflozin and placebo groups. BTMs assessed include BSAP, P1NP, CTX-1 and TRAP-5b. Data are presented as median (IQR) and were analysed using the Wilcoxon signed-rank test for within-group comparisons..

BTMs are presented in Fig. 2, which shows within-group analysis of BTM values before and after 12 months of treatment in both groups. There was a significant decrease in BASP and TRAP-5b in both groups. In contrast, the placebo group showed a statistically significant increase in CTX-1 after 1 year.

After 12 months of treatment, BTM assessment revealed no significant intergroup differences between the dapagliflozin and placebo groups. As shown in Table 3, the adjusted median levels of BALP, total P1NP, CTX-1 and TRAP-5b were comparable between the dapagliflozin and placebo groups.

**Table 3:** Bone turnover markers after 12 months.

Characteristics	Drug, median (IQR)	Placebo, median (IQR)	*P*-value^a^
BSAP (pg/ml)	10.90 (8.96–13.46)	11.48 (9.41–13.23)	.87
P1NP (µg/l)	30.1 (26.2–35.8)	30.6 (26.1–36.2)	.86
CTX-1 (ng/ml)	252.99 (176.9–396.8)	281.46 (201.4–426.5)	.71
TRAP-5b (pg/ml)	426.51 (391.6–489.7)	419.73 (386.1–505.7)	.69

^a^Quantile regression.

**Figure 3:**
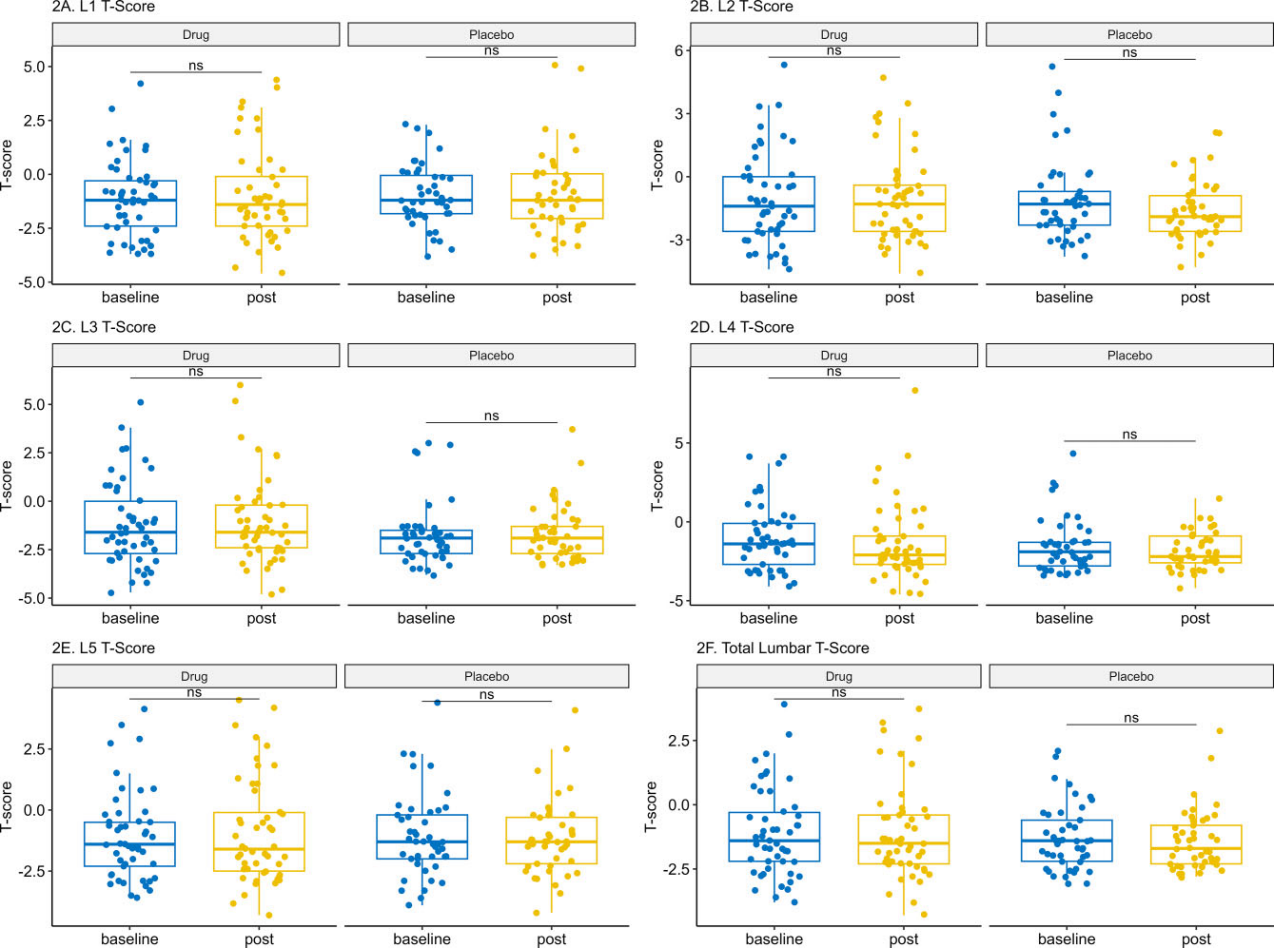
Changes in lumbar spine T-scores. This figure presents the T-scores in the lumbar spine (L1–L5) and total lumbar spine measured by QCT at baseline and 12 months. Data are presented as median (IQR) and analysed using the Wilcoxon signed-rank test for within-group comparisons.

**Figure 4:**
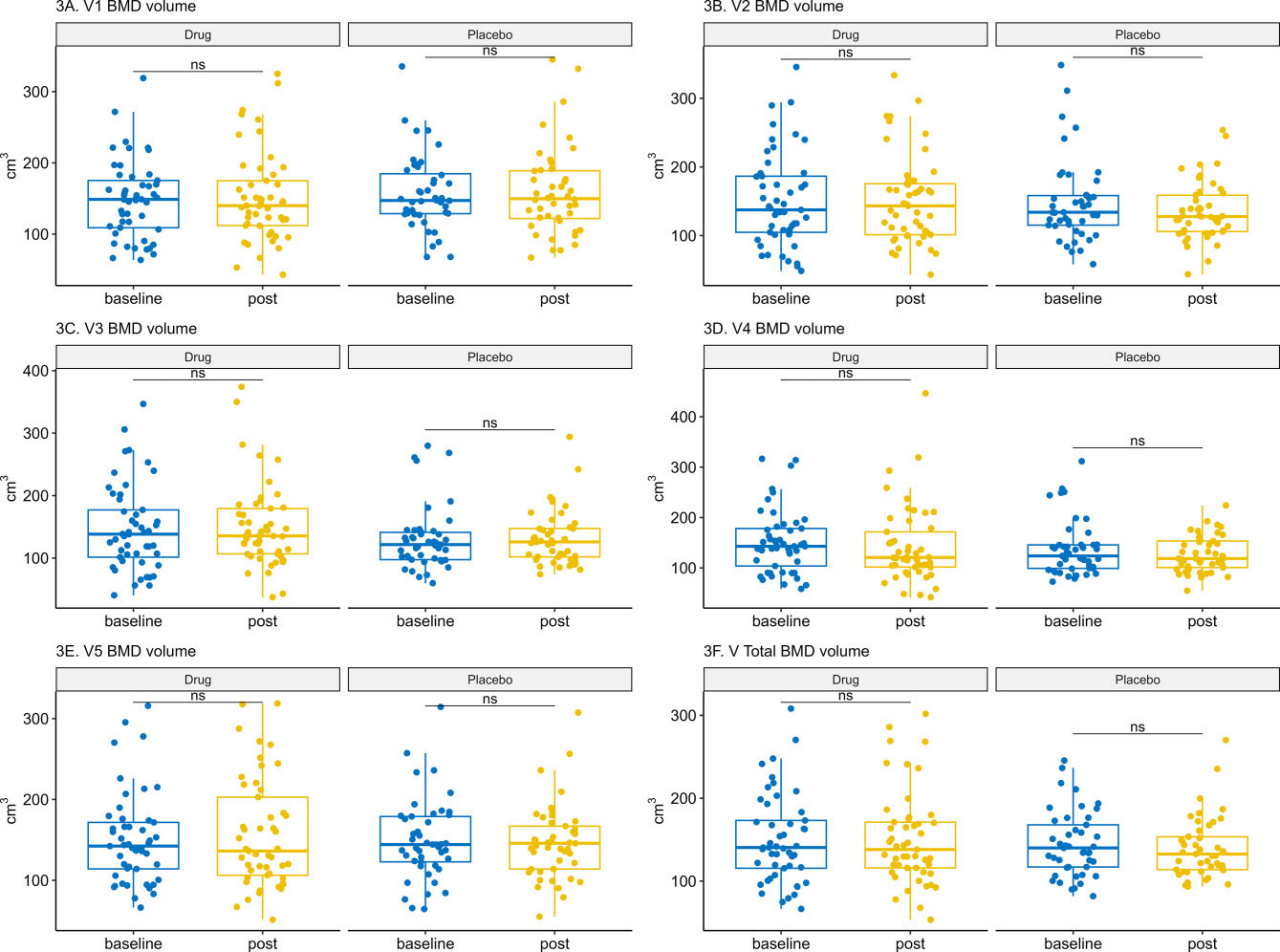
Lumbar spine vBMD changes over 12 months. This figure displays changes in vBMD at different lumbar spine levels (L1–L5) and total lumbar spine measured by QCT at baseline and 12 months. Data are presented as median (IQR) and analysed using the Wilcoxon signed-rank test for within-group comparisons.

At baseline and after 1 year of treatment the T-score at multiple vertebral levels (from L1 to L5) and total T-score were comparable between the dapagliflozin and placebo groups (Fig. 3 and Table 4). Volume measurements of lumbar vertebrae by QCT at baseline and after 12 months of treatment were comparable between the dapagliflozin and placebo groups at different levels and total scores (Fig. 4 and Table 5).

**Table 4:** QCT bone findings (T-score) after 1 year.

Bone	Drug, median (IQR)	Placebo, median (IQR)	*P*-value^a^
L1	−1.08 (−1.84–0.03)	−0.92 (−1.67 to −0.41)	.23
L2	−1.31 (−2.11–0.01)	−1.67 (−2.31 to −0.81)	.08
L3	−1.66 (−2.15 to −0.60)	−1.41 (−2.22–0.85)	.46
L4	−1.89 (−2.49 to −0.71)	−1.67 (−2.24 to −0.91)	.51
L5	−1.08 (−2.07 to −0.09)	−1.18 (−2.31 to −0.40)	.61
L Total	−1.23 (−1.76 to −0.69)	−1.23 (−1.86 to −0.99)	.15

^a^Quantile regression

**Table 5:** QCT bone findings (BMD volume) after 1 year.

Bone	Drug, median (IQR)	Placebo, median (IQR)	*P*-value^a^
L1 (cm^3^)	150.88 (126.85–186.83)	153.93 (130.30–170.73)	.36
L2 (cm^3^)	149.62 (112.72–175.60)	135.63 (112.62–156.12)	.12
L3 (cm^3^)	133.09 (117.14–164.53)	138.38 (114.30–157.47)	.55
L4 (cm^3^)	125.80 (110.42–167.94)	131.44 (112.10–159.53)	.38
L5 (cm^3^)	148.28 (115.59–184.17)	147.81 (113.04–169.95)	.53
L Total (cm^3^)	148.45 (130.13–168.39)	147.53 (127.74–162.46)	.13

^a^Quantile regression.

Multivariable analyses confirmed our primary findings (Supplementary Tables S1–S5). Both DAG-based minimal models and comprehensive clinical models demonstrated no statistically significant differences between the dapagliflozin and placebo groups for any bone outcome (all *P* > .05). The consistency of results across different adjustment strategies strengthens confidence in these findings.

## DISCUSSION

In this 1-year exploratory randomized, prospective, double-blind, placebo-controlled trial, there were no significant differences in BTMs or vBMD between dapagliflozin and placebo in non-diabetic CKD patients. These findings provide preliminary evidence regarding possible bone safety in this understudied population.

Both the dapagliflozin and placebo groups demonstrated a significant decrease in BSAP levels over the 1-year study period (*P* < .001). However, this decrease was not significantly different between groups, suggesting it was not attributable to dapagliflozin treatment. This finding aligns with previous research by Masajtis-Zagajewska *et al.* [20], who reported no impact of empagliflozin use on BSAP alterations in both diabetic and non-diabetic CKD patients. Mirroring the decrease in BSAP, both groups exhibited a decrease in TRAP-5b without statistically significant intergroup differences. This coupled decrease in both bone formation (BSAP) and resorption (TRAP-5b) markers suggests an overall trend toward reduced bone turnover. El-Husseini *et al.* [23] reported that low bone turnover is very prevalent (84%) among patients with mild to moderate CKD. A subgroup analysis of a randomized controlled trial (RCT) on 103 Japanese patients with type 2 diabetes mellitus (T2DM) found that the use of ipragliflozin was associated with an increased level of TRAP-5b after 12 and 24 weeks. This discrepancy in the results might be explained by the difference in the study populations and the follow-up period. On the other hand, the same study showed that BSAP levels did not exhibit significant changes with the use of ipragliflozin, which aligns with our results [11].

In addition to BSAP and TRAP-5b, we assessed other key BTMs. P1NP, a marker of bone formation, after 1 year, showed no significant difference between groups. These results were consistent with findings by Bolinder *et al.* [10], who reported no significant changes in P1NP with dapagliflozin treatment over 102 weeks in European patients with T2DM.

Besides TRAP-5b, another bone resorption marker (CTX-1) showed no significant intergroup differences after 1 year. This finding contrasts with a study done by Bilezikian *et al.* [17], who reported increased CTX levels with canagliflozin. This discordance in the results may be attributed to dissimilarities in study population and different sample size, while our study was a single-centre trial on non-diabetic CKD patients, this study recruited patients with T2DM from 17 different countries. Similar to our results, Bolinder *et al.* [10] found that dapagliflozin did not affect the CTX level after 102 weeks of treatment.

The baseline QCT findings (T-score) for patients in the dapagliflozin and placebo groups showed no statistically significant differences between the two groups. When comparing dapagliflozin with placebo after 1 year, adjusted median T-scores were generally comparable across lumbar levels. On the other hand, the adjusted median vBMD was comparable between both groups. While our study demonstrated stability in BMD parameters over 1 year, another RCT involving patients with T2DM demonstrated a slight but statistically significant decrease in BMD in the total hip area over a 104-week follow-up period in the canagliflozin group [17]. The dissimilarities in the results might be related to differences in the follow-up period as well as in the study population. Conversely, another RCT reported no significant differences in BMD between dapagliflozin and a placebo, as measured by the adjusted mean percent change from baseline at week 102 across the lumbar spine, femoral neck and total hip regions [10]. This finding was supported by a meta-analysis of 20 RCTs, including 12 764 patients, that concluded SGLT2is had no significant impact on lumbar spine BMD in patients with T2DM, aligning with our results [24].

Diabetes mellitus, particularly type 1, may adversely impact bone health and increase fracture risk. While type 1 DM typically lowers BMD due to insulin and insulin-like growth factor-1 deficiencies, T2DM is associated with impaired bone quality. Factors like hyperglycaemia, advanced glycation end-products and inflammation negatively affect bone structure and delay fracture healing, making effective glycaemic control essential to reduce these detrimental effects [25]. It should be noted that this study excluded diabetic patients to rule out any potential effect of diabetes or its glycaemic control on bone metabolism.

Dapagliflozin administration resulted in a statistically significant reduction in both body weight and BMI over the 1-year study period. Participants in the drug group experienced an average weight loss of 2.5 kg (*P* < .05). These findings are consistent with a meta-analysis of 116 RCTs encompassing 98 497 patients that demonstrated a mean weight reduction of 1.79 kg (*P* < .001) with SGLT2i use compared with placebo [26]. Rapid or significant weight loss, particularly in individuals with normal or low BMI, is associated with decreased BMD and an elevated risk of fracture, this is often attributed to a concomitant reduction in lean mass, a crucial determinant of bone strength [27, 28]. On the other hand, several studies have suggested that if the weight loss is primarily fat mass, it is likely to improve BMD relative to body weight [29–31]. Importantly, the weight loss observed in our study with dapagliflozin was relatively modest and occurred gradually over the course of a year. Moreover, the absence of significant changes in BMD parameters, as assessed by QCT, suggests that this degree of weight loss did not adversely impact bone health in this study population.

Many studies have explored the renoprotective effect of SGLT2is, which is thought to be partly achieved by mechanisms independent of lowering blood glucose levels [32]. In this study, upon follow-up 1 year after the intervention, no statistically significant differences were observed in renal function between the dapagliflozin and control groups. In addition, there was a marked reduction of proteinuria in the dapagliflozin group compared with placebo, aligning with the DAPA-CKD trial (NCT03036150) analysis, which demonstrated a 29.3% reduction in proteinuria with dapagliflozin compared with placebo. Notably, this effect was more pronounced in diabetic versus non-diabetic patients (35.1% versus 14.8%) [33]. Furthermore, Cherney *et al.* [34] found that 6-weeks dapagliflozin treatment did not affect proteinuria in non-diabetic patients with CKD. However, Hammad *et al.* [35] found that 3-months of empagliflozin was associated with a greater reduction of proteinuria as compared with placebo in non-diabetic proteinuric patients.

In this study, there was no significant change in urinary calcium excretion after 1 year of treatment; however, it is important to note that the absolute values were low in both groups, suggesting that any potential clinical relevance of this finding might be limited. It should be noted that similar stability in the serum calcium level has been reported in other clinical trials involving SGLT2is. Masajtis *et al.* [20] found that serum calcium and urinary calcium:creatinine ratio were not affected by empagliflozin use in diabetic and non-diabetic patients with CKD, aligning with our results. De Jong *et al.* [36] found that the use of dapagliflozin was not associated with an alteration in serum calcium levels. This finding was confirmed by Bolinder *et al.* [10], who found stability in the serum calcium level after 102 weeks of dapagliflozin. On the other hand, Blau *et al.* [16] demonstrated that 5 days of treatment with canagliflozin 300 mg in healthy volunteers was associated with a significant decrease in urinary calcium excretion without significant changes in serum calcium levels. The discrepancy in the impact of SGLT2is on urinary and serum calcium levels in the Blau *et al.* [16] trial and this study could be attributed to differences in the study populations and the follow-up period.

Dapagliflozin did not exert any impact on serum phosphorus and urinary phosphorus:creatinine ratio after 1 year of treatment in this study. These results contradict Ljunggren *et al.* [19], who showed that dapagliflozin causes a slight increase in serum phosphorus (0.06 mmol/l) after 50 weeks of treatment. Moreover, de Jong *et al.* [36] found that 6 weeks of treatment with dapagliflozin increased serum phosphate by 9% compared with the placebo in 31 patients with diabetic kidney disease. This might stimulate PTH release in their patients, as there was a 16% increase in the serum PTH levels. Of note, the current study did not include any patient with diabetes, which could explain the differences in the results.

There was no significant effect of SGLT2i on iPTH serum levels in this study. This result was different from de Jong *et al.* [36] and Masajtis *et al.* [20], who found a meaningful increase in the serum PTH levels after SGLT2i use. The discrepancy in the results may be attributed to the shorter follow-up period and the patient population. On the other hand, Bolinder *et al.* [10] found that 102 weeks treatment with dapagliflozin was not associated with any change in the PTH level in 140 patients with T2DM. This may suggest that treatment with dapagliflozin is associated with early elevation of PTH levels, which is explained by phosphate retention, followed by normalization of these values.

In this study, the serum magnesium level showed a numerical but non-statistical significance increase in the dapagliflozin group 1 year after the intervention, suggesting a trend toward magnesium retention with SGLT2i use. Furthermore, a meta-analysis of 18 RCTs, comprising 15 309 patients indicated a modest increase in serum magnesium levels among patients with T2DM treated with various SGLT2 inhibitors [37].

This exploratory, hypothesis-generating, double-blind, randomized controlled, single-centre trial evaluated the effects of dapagliflozin on bone health in non-diabetic patients with CKD over a 12-month period. By integrating BTMs and vBMD measured by QCT, the study provides a comprehensive assessment of dapagliflozin’s potential skeletal effects in a population with limited existing evidence. While the findings showed no significant changes in BTMs or vBMD, offering preliminary reassurance regarding bone safety, several limitations should be acknowledged. First, the sample size was modest and based on effect size estimates derived from canagliflozin studies, as no dapagliflozin-specific data were available at the time of study design. Other research suggests that dapagliflozin may exert smaller or negligible effects on BTMs, indicating that the initial assumptions may have been optimistic. Second, although β-CTX concentrations can be influenced by kidney function, this effect becomes clinically relevant primarily in advanced CKD, particularly when eGFR falls below 30 ml/min/1.73 m^2^ [38]. In our cohort, only a small number of participants had an eGFR <30 ml/min/1.73 m^2^, making substantial GFR-related variability unlikely. Moreover, we further minimized potential confounding by stratifying randomization according to baseline eGFR and by incorporating kidney function into both minimal DAG-based models and comprehensive multivariable adjustment analyses. The consistency of findings across these complementary analytic approaches enhances the robustness and reliability of our results. Finally, the 12-month follow-up period may not have been sufficient to detect long-term or microarchitectural skeletal effects, which could be better assessed using high-resolution peripheral QCT or bone biopsy in future studies.

## Supplementary Material

sfaf384_Supplemental_Files

## Data Availability

The data that support the findings of this study are available from the corresponding author upon reasonable request.
