# Supplementary material for: Impact of dapagliflozin on bone mineral metabolism in non-diabetic patients with chronic kidney disease: a randomized, double-blind, placebo-controlled study
Source: Clin Kidney J. 2025 Dec 9;19(3):sfaf384. doi: 10.1093/ckj/sfaf384 (PMC12963970; doi:10.1093/ckj/sfaf384)
Supplement: sfaf384_Supplemental_Files [file sfaf384_supplemental_files.zip › Supplementary tables.docx]

Table S1. Comprehensive Multivariable Models: QCT Measures

|  | **vBMD (cm³)** | | | **T-score** | | |
| --- | --- | --- | --- | --- | --- | --- |
|  | **vBMD**^1^ | **95% CI**^1^ | **p-value**^1^ | **T-score**^1^ | **95% CI**^1^ | **p-value**^1^ |
| Placebo | — | — |  | — | — |  |
| Drug | 9.1 | -1.6, 20 | 0.094 | 0.26 | -0.07, 0.60 | 0.12 |
| ^1^Adjusted for baseline eGFR, baseline QCT values, age, sex, weight change, baseline PTH, BMI, and CKD etiology | | | | | | |
| Abbreviation: CI = Confidence Interval | | | | | | |

Table S2. DAG-Based Multivariable Models: Bone Turnover Markers

|  | **BSAP (µg/L)** | | | **P1NP (µg/L)** | | | **CTX-1 (ng/ml)** | | | **TRAP-5b (pg/ml)** | | |
| --- | --- | --- | --- | --- | --- | --- | --- | --- | --- | --- | --- | --- |
|  | **BSAP**^1^ | **95% CI**^1^ | **p-value**^1^ | **P1NP**^1^ | **95% CI**^1^ | **p-value**^1^ | **CTX-1**^1^ | **95% CI**^1^ | **p-value**^1^ | **TRAP-5b**^1^ | **95% CI**^1^ | **p-value**^1^ |
| Treatment |  |  |  |  |  |  |  |  |  |  |  |  |
| Placebo | — | — |  | — | — |  | — | — |  | — | — |  |
| Drug | -0.14 | -2.5, 2.2 | >0.9 | -0.39 | -5.9, 5.1 | 0.9 | -40 | -108, 29 | 0.3 | -6.5 | -66, 53 | 0.8 |
| Baseline GFR | 0.00 | -0.10, 0.09 | >0.9 | 0.14 | -0.09, 0.37 | 0.2 | -0.56 | -3.4, 2.3 | 0.7 | 1.1 | -1.4, 3.5 | 0.4 |
| ^1^Adjusted for baseline eGFR only (minimal sufficient set from DAG analysis) | | | | | | | | | | | | |
| Abbreviation: CI = Confidence Interval | | | | | | | | | | | | |

Table S3. DAG-Based Multivariable Models: QCT Measures

|  | **vBMD (cm³)** | | | **T-score** | | |
| --- | --- | --- | --- | --- | --- | --- |
|  | **vBMD**^1^ | **95% CI**^1^ | **p-value**^1^ | **T-score**^1^ | **95% CI**^1^ | **p-value**^1^ |
| Treatment |  |  |  |  |  |  |
| Placebo | — | — |  | — | — |  |
| Drug | 9.5 | -10, 29 | 0.3 | 0.31 | -0.32, 0.94 | 0.3 |
| Baseline GFR | 0.75 | -0.08, 1.6 | 0.077 | 0.02 | 0.00, 0.05 | 0.080 |
| ^1^Adjusted for baseline eGFR only (minimal sufficient set from DAG analysis) | | | | | | |
| Abbreviation: CI = Confidence Interval | | | | | | |

Table S4. Comprehensive Multivariable Models: Bone Turnover Markers

|  | **BSAP (µg/L)** | | | **P1NP (µg/L)** | | | **CTX-1 (ng/ml)** | | | **TRAP-5b (pg/ml)** | | |
| --- | --- | --- | --- | --- | --- | --- | --- | --- | --- | --- | --- | --- |
| **Characteristic**^1^ | **BSAP**^1^ | **95% CI**^1^ | **p-value**^1^ | **P1NP**^1^ | **95% CI**^1^ | **p-value**^1^ | **CTX-1**^1^ | **95% CI**^1^ | **p-value**^1^ | **TRAP-5b**^1^ | **95% CI**^1^ | **p-value**^1^ |
| Placebo | — | — |  | — | — |  | — | — |  | — | — |  |
| Drug | 0.01 | -2.6, 2.6 | >0.9 | 1.3 | -4.9, 7.6 | 0.7 | -64 | -146, 18 | 0.13 | 2.0 | -66, 70 | >0.9 |
| ^1^Adjusted for baseline eGFR, baseline bone markers, age, sex, weight change, baseline PTH, BMI, and CKD etiology | | | | | | | | | | | | |
| Abbreviation: CI = Confidence Interval | | | | | | | | | | | | |

| **Table S5: Comparison of DAG-based vs Comprehensive Multivariable Models** | | | | |
| --- | --- | --- | --- | --- |
| **Treatment Effects: Dapagliflozin vs Placebo** | | | | |
|  | **DAG-Based Models** | | **Comprehensive Models** | |
| **Outcome** | **β (95% CI)** | **p-value** | **β (95% CI)** | **p-value** |
| BSAP | -0.14 (-2.46, 2.18) | 0.905 | 0.01 (-2.57, 2.58) | 0.996 |
| P1NP | -0.39 (-5.91, 5.12) | 0.888 | 1.34 (-4.91, 7.59) | 0.670 |
| CTX-1 | -39.78 (-108.45, 28.89) | 0.252 | -63.60 (-145.65, 18.45) | 0.127 |
| TRAP-5b | -6.53 (-65.66, 52.59) | 0.827 | 2.00 (-65.80, 69.80) | 0.953 |
| vBMD | 9.52 (-10.06, 29.10) | 0.337 | 9.09 (-1.59, 19.76) | 0.094 |
| T-score | 0.31 (-0.32, 0.94) | 0.337 | 0.26 (-0.07, 0.60) | 0.124 |
| DAG Model: Adjusted for baseline eGFR only. Comprehensive Model: Adjusted for baseline eGFR, baseline outcome values, age, sex, weight change, baseline PTH, BMI, and CKD etiology. | | | | |
