# Supplementary material for: Impact of dapagliflozin on bone mineral metabolism in non-diabetic patients with chronic kidney disease: a randomized, double-blind, placebo-controlled study
Source: Clin Kidney J. 2025 Dec 9;19(3):sfaf384. doi: 10.1093/ckj/sfaf384 (PMC12963970; doi:10.1093/ckj/sfaf384)
Supplement: sfaf384_Supplemental_Files [file sfaf384_supplemental_files.zip › Supplementry figure.docx]

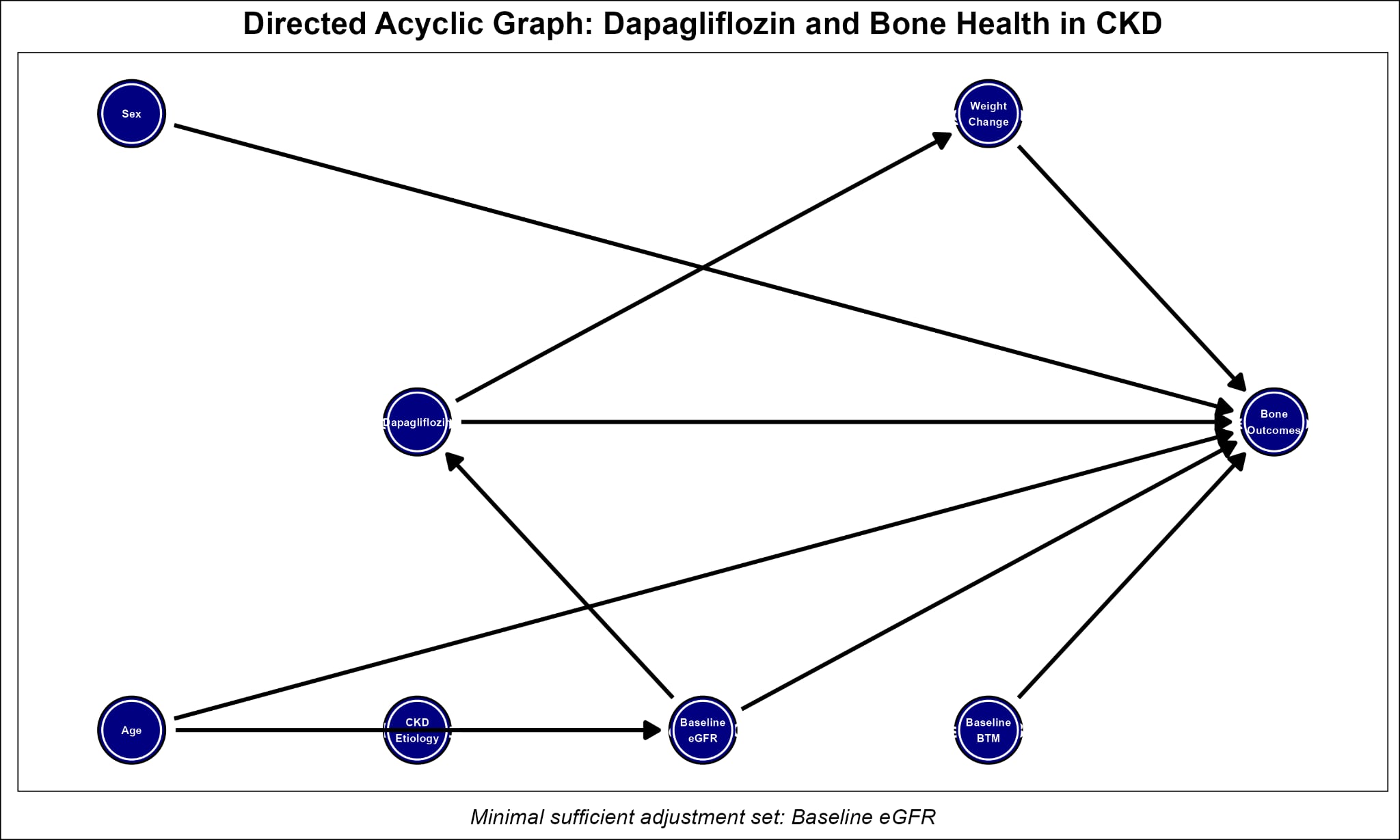


**Supplementary Figure 5. Directed Acyclic Graph (DAG) for the effect of dapagliflozin on bone health in CKD.**
This DAG illustrates the hypothesized relationships between dapagliflozin use and bone outcomes in patients with chronic kidney disease (CKD). Potential confounders include age, sex, CKD etiology, baseline bone turnover markers (BTM), and baseline estimated glomerular filtration rate (eGFR). Weight change is depicted as a potential mediator. Based on DAG analysis, the minimal sufficient adjustment set to control for confounding was baseline eGFR.
